# Formulation and Particle Size Reduction Improve Bioavailability of Poorly Water-Soluble Compounds with Antimalarial Activity

**DOI:** 10.1155/2013/769234

**Published:** 2013-05-12

**Authors:** Hongxing Wang, Qigui Li, Sean Reyes, Jing Zhang, Lisa Xie, Victor Melendez, Mark Hickman, Michael P. Kozar

**Affiliations:** Department of Drug Discovery, Division of Experimental Therapeutics, Walter Reed Army Institute of Research, 503 Robert Grant Avenue, Silver Spring, MD 20910, USA

## Abstract

Decoquinate (DQ) is highly effective at killing malaria parasites *in vitro*; however, it is extremely insoluble in water. In this study, solid dispersion method was used for DQ formulation which created a suitable physical form of DQ in aqueous phase for particle manipulation. Among many polymers and surfactants tested, polyvinylpyrrolidone 10, a polymer, and L-**α**-phosphatidylcholine or polysorbate, two surfactants, were chosen as DQ formulation components. The formulation particles were reduced to a mean size between 200 to 400 nm, which was stable in aqueous medium for at least three weeks. Pharmacokinetic (PK) studies showed that compared to DQ microparticle suspension, a nanoparticle formulation orally dosed to mice showed a 14.47-fold increase in area under the curve (AUC) of DQ plasma concentration and a 4.53-fold increase in AUC of DQ liver distribution. WR 299666, a poorly water-soluble compound with antimalarial activity, was also tested and successfully made into nanoparticle formulation without undergoing solid dispersion procedure. We concluded that nanoparticles generated by using appropriate formulation components and sufficient particle size reduction significantly increased the bioavailability of DQ and could potentially turn this antimalarial agent to a therapeutic drug.

## 1. Introduction

DQ has been marketed as a veterinary medicine for inhibiting the growth of coccidiosis in the digestive system of poultry for many years without any obvious adverse effects [1]. This compound has been shown experimentally to have efficacy against diarrheal disease caused by *Cryptosporidium parvum *[2, 3]. It has also been shown to be highly effective against malaria parasites in both the blood and liver stages as shown in some rodent and primate malaria models [4–8]. Unlike the classic antimalarial drugs such as chloroquine, which enters the red blood cells, is selectively accumulated in the *Plasmodium* lysosome, inhibits hematin body packaging, and forms highly toxic complex to the parasites by chloroquine and hematin binding, DQ has recently emerged as a potent *in vitro* and *in vivo* inhibitor of the *Plasmodium* liver stage and acts by selectively and specifically inhibiting the parasite's mitochondrial electron transport chain [6]. DQ exhibited minimal cross-resistance with its analog compound atovaquone, an existing antimalarial drug. Although both DQ and atovaquone inhibit cytochrome bc1 complex, there is evidence that they have distinctly different modes of binding within the ubiquinol-binding site of cytochrome b [6]. DQ has also shown to have potent activity against developing gametocytes, the parasite transmission form, which is additional evidence that the two drugs interact differently with the ubiquinol-binding site [7]. The insolubility of DQ in water has limited its broad use as a systemic treatment agent for infectious diseases such as malaria and cryptosporidiosis. Currently this compound has not been developed as an active component for treating or preventing malaria in humans or animals. 

DQ, a 4-hydroxyquinoline, is highly lipophilic and its water solubility is exceptionally low [9]. Water insoluble drugs which exhibit excellent *in vitro* potency may be converted to useful therapeutic agents if sufficient improvement of bioavailability can be achieved. Drug microparticles and nanoparticles have been shown to significantly improve drug permeability, bioavailability and enhance drug exposure for oral and parenteral dosage forms. This is based on previous data showing that reduction of drug crystals in size from 10 microns to 100 nm particles generates a 100-fold increase in surface area to volume ratio [10]. This increase in surface area has a profound impact on the dissolution rate and absorption of the molecule. The increased dissolution rate can significantly improve the performance of poorly water-soluble compounds. 

There are several techniques that are known to produce drug microparticles and nanocrystals and to improve the dissolution rate of compounds with poor oral bioavailability [11]. One of the current methods used to generate drug nanoparticles is by high pressure homogenization (HPH) [12]. Typically, drug microparticles and nanocrystals are generated in a liquid dispersion medium (e.g., by precipitation or a disintegration process). The product obtained from this process is a suspension of small drug particles in a liquid stabilized by a surfactant or polymer [13–15]. In contrast to micronized powders, drug nanocrystals can be administered using very different administration routes [10]. Solid dispersion is also an important approach for improvement of bioavailability of poor water-soluble drugs [15, 16]. It is a commonly used method for formulation of insoluble drug molecules scattered in other water-soluble materials in a solid state prior to the addition of water. Recent reports in the literature have demonstrated the benefits in improved bioavailability by formulating drugs with reduced particle size. The antimalarial agent, halofantrine, was formulated into various solid dispersions, and the reformulated dispersions showed a five- to seven-fold increase in absolute bioavailability [17]. Other examples of drugs that have benefited from the reduction of drug particle sizes leading to increased bioavailability have also been reported [18–21].

In this study, we have made DQ in nanoparticle formulations by solid dispersion, use of formulation carriers, and particle size reduction to increase DQ aqueous compatibility and bioavailability. The formulation carriers included polymers such as PVP, specifically PVP 10, and surfactants such as PC or polysorbate 80 (Tween 80) or both. The particle size of DQ formulation was aimed to achieve nanometer range to improve its bioavailability. A water insoluble compound, WR299666, previously synthesized by our institute, has powerful prophylactic activity against *Plasmodium falciparum in vitro* and in mice against *Plasmodium berghei* via intramuscular injection. It was made in various particle sizes as an example for comparison with DQ to evaluate different techniques used to produce microparticle and nanoparticle drugs. Various preparations of these compounds were administered to mice by PO (manually intragastric method) and their pharmacokinetic profiles studied. The drug concentrations in the plasma and the liver were determined by LC-MS/MS (Liquid Chromatography/Mass Spectrometry) assay and analyzed with PK modeling software. 

## 2. Materials and Methods

### 2.1. Reagents and Animals

DQ, polyvinylpyrrolidone (PVP 10 and providence), dextrose, egg L-*α*-phosphatidylcholine (PC), polysorbate (Tween 80), and hydroxyethyl cellulose (HEC) were purchased from Sigma, Saint Louis, Missouri. Poloxamer 407 was obtained from Spectrum Chemical MFG Corporation (New Brunswick, NJ). Methanol, n-butyl chloride, ethanol, and all other reagents used in the study were of the highest purity commercially available. WR299666 was synthesized in this institute, and the structure is shown in Figure 2. Male ICR mice obtained from Charles River Laboratories were used in this study. On arrival, the animals were acclimated for 7 days (quarantine). Mice were 7 weeks of age upon the initiation of dosing. The animals were housed singly in a cage maintained in a room with a temperature range of 18–26°C, 34–68% relative humidity and a 12-hour light/dark cycles. Food and water were provided *ad libitum* during quarantine and throughout the study. The animals were fed a standard rodent maintenance diet. Research was conducted in compliance with the Animal Welfare Act and other federal statutes and regulations relating to animals and experiments involving animals and adheres to principles stated in the *Guide for the Care and Use of Laboratory Animals*, NRC publication, 2011 edition.

### 2.2. Drug Suspension Preparation

The suspension of DQ in HECT (0.5% hydroxyethyl cellulose (w/v) and 0.2% Tween 80 (v/v) in distilled water) was predispersed for 5 minutes using a Misonix Ultrasonic Liquid Processor Q 500 (Q Sonica, LLC., Newtown, CT) at 15% amplitude with a 7 mm diameter probe. The unit was then programmed to sonicate at 25% amplitude for another 5 minutes, shut off for 2 minutes, and then re-started at 15% amplitude for 2 minutes. A comparison of DQ homogenization techniques was also conducted utilizing a homogenizer equipped with a 30 mm open slotted generator running at 20000–22000 rpm for 5 minutes. Without homogenization or sonication, DQ particles were not suspended evenly in water or HECT, and the particle size of the raw material could not be measured accurately. The information on the particle size was not available. 

The suspension of WR299666 was also prepared in HECT in distilled water, using a homogenizer (PRO Scientific Inc. Monroe, CT) with a 30 mm open slotted generator to homogenize the drug powder mixture at 20000–22000 rpm for 5 minutes. The particle size of the starting raw material of WR 299666 was above 70 *μ*m in average without treatment. Homogenization was repeated for one minute intervals in an ice bath until a stable mean particle size value was obtained for each sample. A sonication instrument (Sonics Vibra Cell Sonicator) was subsequently utilized in a cycle of 5-second pulses and 5-second standbys for a total of 20 minutes in an ice bath to further reduce the particle size of the drug in the suspension.

The particle size was measured using a Horiba LA-950 particle size distribution analyzer (Kyoto, Japan), based on laser scattering. This particular instrument has a measurement period of less than 2 minutes from introduction of sample to data presentation with a sample requirement for testing of 2–5 mg per measurement in a 150 mL flow cell, depending on the sample type. All DQ and WR2996666 preparations were measured at a range of transmittance between 80–90%. Various volumes ranging from 100 *μ*L to 800 *μ*L of samples with about 5–10 mg solid from each vial were tested in this instrument to determine the mean particle size, which was the particle size parameter referred in the results. 

### 2.3. DQ Nanoparticle Preparation

DQ nanoparticles were prepared by solid dispersion of DQ with polymers and surfactants followed by particle size reduction using sonication and high pressure homogenization (HPH). Prior to solid dispersion, DQ, poly vinyl pyrrolidone (PVP 10 or povidone), and surfactants of either PC or Tween 80 or both, was individually dissolved in a mixture of ethanol and n-butyl chloride (5 : 3). Each component was then added to the already dissolved DQ solution according to the formulation design. The solvents were removed by rotary evaporation or dried under vacuum. The dried materials were hydrated with water and particle size reduction was performed. 

Such formulated DQ suspended in water was sonicated (Elma S 40H, Singen, Germany) for about 5–30 minutes or until the particle size reduced to less than 10 *μ*m. The suspension was then homogenized by HPH in high pressure homogenizer (Nano DeBEE, BEE international, Inc. South Easton, MA) at a drop of 1500–2500 bars and a reflux temperature of about 30°C. Continuous cold water flow was adopted for cooling the samples. The particle size was measured periodically and the HPH process continued until the particles could not be further reduced. The piston pump stroke frequency utilized was 30 minutes, followed by a pause of 30 minutes, and the entire process was run for a total of 3–6 hours, which is equivalent to a total of 70–190 passes or homogenization cycles. 

The resulting samples with nanoparticles were fast freeze dried in dry ice, soaked in acetone, and lyophilized to powder or thin film under high vacuum at low temperature (−91°C) by using a lyophilizer (Lyo-Centre, Virtis, Gardiner, NY). The lyophilized materials were stored at 4°C for future use or reconstituted with water or normal saline (0.9% NaCl) for further study. 

### 2.4. WR299666 Nanoparticle Preparation

A suspension of WR299666 was predispersed utilizing an Ultrasonic Liquid Processor Q 500. The sample with particles less than 10 *μ*m was suspended in 30–40 mL of HECT and stirred for 10 minutes to achieve satisfactory dispersion. The suspension was then homogenized with a high pressure homogenizer (HPH) set at a drop of 2000 bar for each pass (1 bar = approx 14.5 psi) and a reflux temperature of about 30°C. The piston pump stroke frequency duration was 20 minutes, followed by a pause of 10 minutes, and the process was then run for a total of 1 hour, which was equivalent to a total of 10–15 passes or homogenization cycles. 

### 2.5. Evaluation of Drug Preparations

The rehydrated DQ nanoparticle formulations were evaluated by particle size measurement, determination of drug concentration in the whole suspension as well as in the fine particles less than 0.2 *μ*M, and PK study in drug dosed animals. The particle size was monitored throughout the preparation procedure and before and after animal studies. To ensure that there was no change of the drug integrity in any of the formulation procedure, drug concentrations and chromatography profiles of the drug molecule identity were examined by an Agilent HPLC (Agilent Technologies, Foster City, CA) with an isocratic mobile phase of 20% water and 80% acetonitrile (0.1% formic acid contained in water and acetonitrile, resp.). All drug preparations in aqueous system were diluted in methanol with vigorous vortex and then in acetonitrile for HPLC analysis. This was done to obtain complete extraction of the compound prior to loading the samples to HPLC. To evaluate the amount of DQ in the fine particles, formulation suspensions were prepared in the amount of 10 mg/mL of DQ in water, incubated at 37°C for 2 hours and passed through a 0.2 *μ*M size filter. The filtrates were used for HPLC analysis following the same dilution steps as above. In order to determine the actual dosage of the drug for animal studies, the concentration of the drug used for dosing animals was determined by HPLC without prior particle size fractionation with filtration. 

### 2.6. PK Studies

PK studies were performed using single oral administration. For each time point to be acquired, five 6-week old male ICR mice at 28–32 g body weight were utilized. The animals were dosed at 50 mg/kg for WR299666 with mean particle diameter of 1.5 *μ*M or 80 mg/kg for DQ with nanoparticle size of 0.39 *μ*M. In order to obtain valid values of drug concentrations for microsuspension of DQ with particle size of 36.88 *μ*M, the dosage was increased to 400 mg/kg due to much less efficient absorption of the drug in micronized particles than in nanoparticles. Both drug suspensions were dosed at 100 *μ*L/20 g. After dosing, plasma and liver samples were collected at each time point. The whole blood was collected by cardiac puncture. Blood samples were collected in lithium heparin tubes within 0 h (baseline) prior to drug administration and at 0.5, 1, 2, 4, 8, 24, 48, 72, and 96 hours after drug administration. Following the separation of appropriate aliquots, plasma was obtained from the whole blood *via* centrifugation. All liquid and tissue samples were immediately preserved on dry ice and stored at −80°C until analytical work was performed for LC-MS/MS analysis.

### 2.7. LC-MS/MS Assay

Mouse plasma samples (100 *μ*L) were analyzed for WR299666 and DQ using an LC-MS/MS system. The samples were separated on a Waters Xterra MS C_18_ column (3.5 *μ*m particle size, 2.1 × 50 mm) using 5 mM ammonium acetate (pH 4): acetonitrile : water gradient driven by a Surveyor HPLC pump. Mass spectrometric detection was accomplished on a ThermoFinnigan TSQ AM triple quadrupole mass spectrometer (Vernon Hills, Illinois) equipped with an electrospray ionization source. Standard curve and quality control (QC) samples were generated by spiking interference-free mouse plasma samples with known amounts of DQ or WR299666 and an internal standard. The drug concentrations of the QC samples chosen were within the range of the standard curve and included a lower limit of quantification (LLOQ), with low (<3x LLOQ), medium, and high QC levels. The validated concentration range of the method was 4 ng/mL to 800 ng/mL for WR299666 and DQ. Individual samples were prepared for LC-MS/MS analysis using a protein precipitation method. Spiked mouse plasma samples (100 *μ*L) were diluted with an equal volume of cold acetonitrile containing indomethacin as an internal standard. Plasma proteins were then precipitated by centrifugation at 15000 rpm for 5 minutes. Ten microliter aliquots of reconstituted standard curve and QC samples were then injected into the LC-MS/MS system for analysis. 

Blank liver homogenate was prepared by adding 5 mL of water for each gram of liver, and then the mixture was homogenized using an ultrasonication probe (Sonics and Material, Inc. Newtown, CT). Plasma and liver homogenate standard curves with added drug compound were prepared *in vitro* via serial dilutions from a high concentration (500 ng/mL) to a low concentration (0.1 ng/mL) with a series of ten to eleven points. The serial dilutions also included 4-5 QC samples corresponding to the high, middle, and low levels of standard curves ranging generally from 10 ng/mL to 100 ng/mL. Once the standard curve dilutions were established, a 100 *μ*L aliquot of plasma or liver samples was removed and extracted with 200 *μ*L acetonitrile. The extracted samples were centrifuged at 10,000 rpm for 10 minutes and the supernatant was removed for analysis by LC-MS/MS. Sample drug concentrations were first interpolated from the standard curve and then multiplied by a factor of 6 to present actual drug levels in the liver prior to dilution with water for homogenization.

Chromatography was performed using a Surveyor pump (Thermo Scientific, Waltham, MA) with Waters XTerra MS C_18_50 mm × 2.1 mm id, 3.5 *μ*m particle size columns (Waters Corp., Milford, MA). Mobile phase consisted of water containing 0.1% formic acid (solvent A) and acetonitrile with 0.1% formic acid (solvent B) gradient. The gradient was initiated at 2% B, increased to 98% B over a period of 1 minute to 3.5 minutes, held steady for 2 minutes, returned immediately to the starting composition, and then allowed to equilibrate for 1.5 minutes. Flow rate was 300 *μ*L/min. Samples were injected using an HTC PAL autosampler (LEAP Technologies, Carrboro, NC). Tandem mass spectrometry was performed using a TSQ Quantum AM (Thermo Scientific). 

### 2.8. Data Analysis

Drug concentrations in QC samples and experimental mouse plasma and liver samples were calculated by a best-fit equation and the PARs obtained from the LC-MS/MS analysis. Calibration standards and QC samples were analyzed to evaluate the performance of the assay. For determination of pharmacokinetic parameters, the concentration-time data of DQ or WR299666 collected during the first day and second day were fitted to a noncompartment analysis (Phoenix/Win Nolin 6.1, Scientific Consulting, Inc. Apex, North Carolina). The AUC was determined by the linear trapezoidal rule with extrapolation to infinity based on the concentration of the last time point divided by the terminal rate constant. 

## 3. Results

DQ nanoparticle formulations were prepared by the procedure involving solid dispersion of the drug with formulation carriers, resuspension in aqueous solution, and particle size reduction by sonication as well as HPH. To ensure that the formulation process and HPH had not been destroyed or changed the DQ molecule, chromatography profiles such as mass quantity and retention time of drug compounds were analyzed by HPLC upon the completion of all preparations. The results showed that the DQ in any of the preparations including microparticles and nanoparticles had no change in chromatography profile which may indicate the integrity of its chemical structure. 

### 3.1. The Particle Size and Stability of Microparticles and Nanoparticles

**Figure 1:**
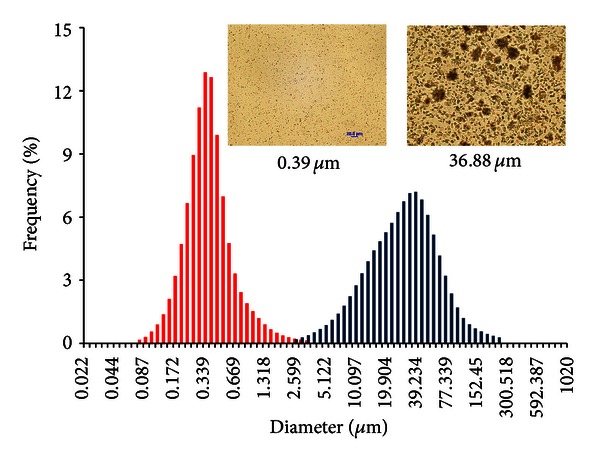
Particle size measurements and microscope observations of DQ nanoparticles made by HPH and suspended in water with a mean diameter of 0.39 *µ*m ((Left, red peak) (*n* = 3)), and DQ microparticles generated by probe ultrasonication and suspended in HECT with a mean particle size of 36.88 *µ*m (Right, dark blue peak) (*n* = 3).

**Figure 2:**
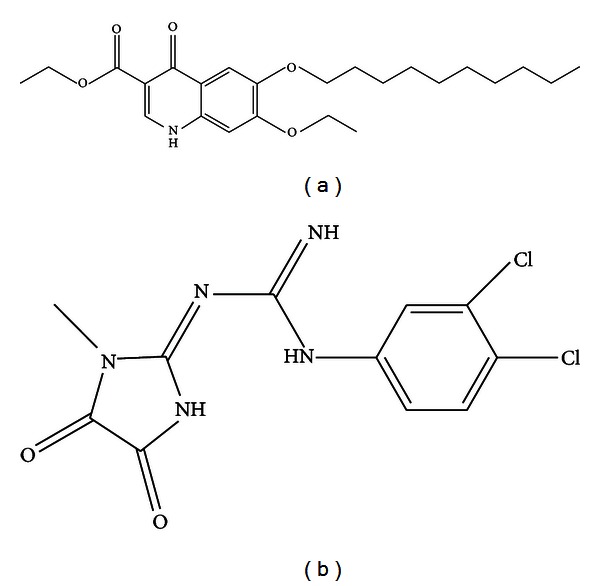
(a) Decoquinate, a 4-hydroxyquinoline (ethyl 6-decyloxy-7-ethoxy-4-hydroxy-3-quinolinecarboxylate), C24H35NO5, molecular weight 417.54, CAS Number 18507-89-6; it is poorly soluble in water (0.06 mg/L in purified water and <0.01 mg/L in buffered water pH 4–9). (See [9]). (b) WR 299666, (E)-1-(3, 4-dichlorophenyl)-3-(1-methyl-4, 5-dioxoimidazolidin-2-ylidene) guanidine, C11H9Cl2N5O2, molecular weight 314.13.

For all preparations, the particle size was measured by the same method using Horiba Instrument. For DQ suspension preparation, 10 minute-sessions of probe sonication were applied to minimize particle size to 36.88 *μ*m. This direct sonication with a probe method worked more efficiently for DQ in HECT than the homogenizer with an open slotted generator which yielded a mean particle size of 102.04 *μ*m (Table 1). The particle size of DQ formulations prepared with combined methods of solid dispersion, use of polymers, and surfactants was reduced to 0.22–0.39 *μ*m (Figures 1, 6, and 7) by sonication followed by HPH at a pressure between 1500–2500 bars for 120 homogenization cycles. 

**Figure 3:**
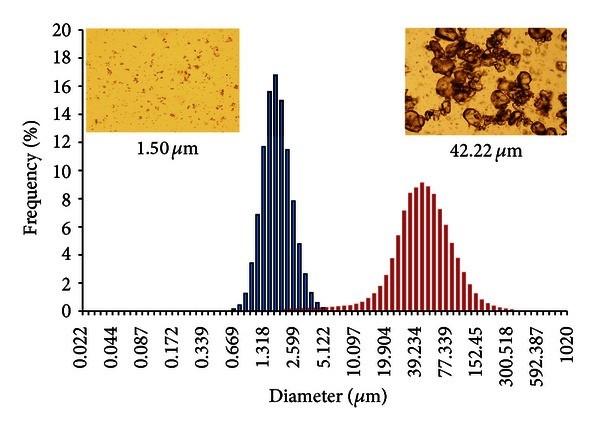
Particle size measurements and microscope observations of WR299666 suspensions made by manual glass grinder with peak in pink (42.22 *µ*m) (*n* = 3) and by ultra-sonicator with peak in blue (1.50 *µ*m) (*n* = 3), both suspended in HECT.

For WR 299666 suspension preparation, 3 minute-sessions of grinding were applied to minimize the particle sizes with a mean diameter of 42.22 *μ*m (Table 2). A procedure with sonicator was performed to evenly distribute pulses of sound to break apart particles. This sonication method produced particles of 1.5 *μ*m for WR299666 which was used in animal studies (Figure 3). To further reduce the particle size of this compound, HPH was applied at a pressure between 1500–2500 bars in 10–15 homogenization cycles. This procedure reduced the particle size of WR299666 to 0.38–0.48 *μ*m under production conditions of 2500 bars in 6 homogenization cycles. 

In order to see the change of particle size of various preparations over time, an accelerated stability study of prepared micro- and nanosuspensions were carried out at a temperature of 4°C for a period of up to 3 weeks. Accurately weighed amounts of samples were placed into glass vials with aluminum-lined caps and stored in a microprocessor-controlled humidity chamber, and the samples were then characterized as a function of exposed time. The stability of particles shown in Tables 1 and 2 was some of the preparations evaluated in this study. Generally, the particles in aqueous suspension dosed in our animals were stable for at least 3 weeks.

### 3.2. DQ Concentrations in the Plasma and the Liver

The oral delivery of DQ with various preparations were compared in animal studies and the drug concentrations at different time points were measured in the plasma and liver samples from animals following oral administration. The data from one study were summarized and shown in Table 3. The values of plasma AUC and *C*_max⁡_ were 795.48 ng·h/mL and 25.3 ng/mL, respectively, for DQ nanoparticle preparation with a mean particle size of 0.39 *μ*m, and 51.37 ng·h/mL and 6.10 ng/mL, respectively, for DQ microsuspension with particle size of 36.88 *μ*m. When the AUC measured in plasma from animals treated with the nanoparticle formulation (0.39 *μ*m) was set as 100%, the AUC in mice treated with DQ suspension in microparticle size (36.88 *μ*m) was only 6.77%, indicating a significant improvement in bioavailability for DQ nanoparitcle formulation reflected by a 14.47-fold increase in the AUC. 

**Figure 4:**
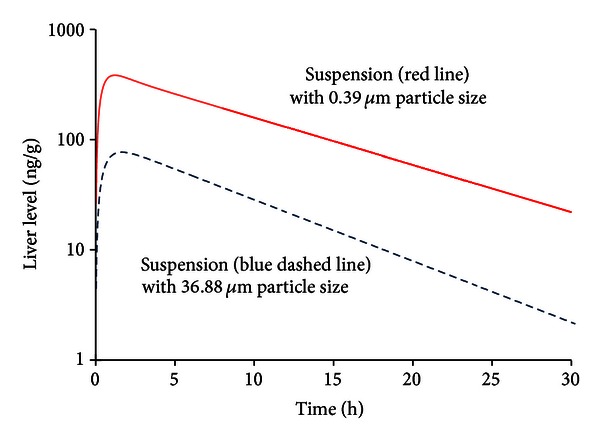
Concentration versus time profiles of DQ in mice liver following single oral dose of nanoparticles in aqueous solution (0.39 *µ*m, red solid line) and microparticle suspension in HECT in distilled water (36.88 *µ*m, blue dashed line).

The liver-distribution data indicated that the AUC and *C*_max⁡_ of DQ in the liver were 6055 ng·h/g and 407.26 ng/g, respectively, in mice treated with the nanoparticle formulation and 1096 ng·h/g and 93.38 ng/g, respectively, in the mice treated with the micronized particles (Table 3). Based on the AUC value, the DQ distribution in the liver was 4.53-fold higher in nanoparticles than in microparticles. When the relative drug distribution in animal livers treated with the nanoparticle formulation was set as 100%, the relative drug distribution in the livers of mice treated with the DQ microparticles was only 18.10% (Figure 4). These results suggested that the liver distribution of DQ in mice was dramatically increased with the use of DQ nanoparticle formulation. 

### 3.3. Relative Distributions of DQ in the Liver and the Blood

In comparison of relative DQ distributions in the plasma and in the liver, both groups of animals, respectively, treated with nanoparticles and micronized particles demonstrated significantly much higher concentration of DQ in the liver than in the plasma. As can be seen in Table 4 which summarized the data obtained from PK analysis, the DQ values of the AUC and *C*_max⁡_ in the liver of the nanoparticle formulation group were 81.20-fold and 16.10-fold of those in the plasma, whereas these values in the liver of the DQ microparticle suspension group were 21.30-fold and 15.30-fold that of plasma levels. The ratios of *C*_max⁡_ in the liver versus in the plasma for both groups were similar but the ratio of AUC in the liver versus in the plasma in the nanoparticle group was much higher than in the microparticle suspension group, indicating higher DQ enrichment in the liver for nanoparticle formulations than for microsuspension. 

### 3.4. Relative Bioavailability of WR299666 Mircroparticles

**Figure 5:**
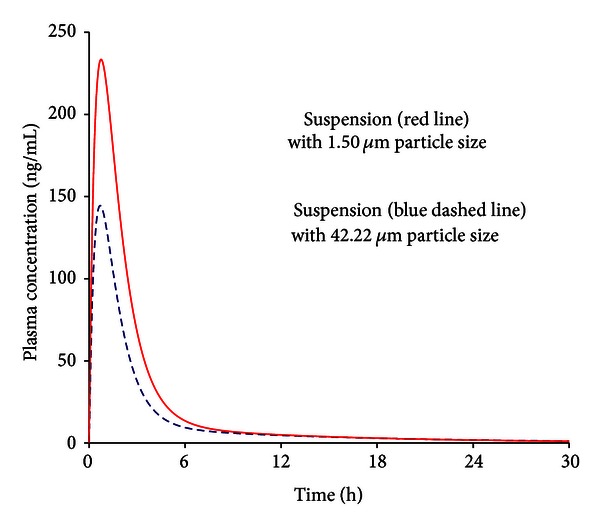
Concentration versus time profiles of WR299666 in plasma following single oral dose of suspensions in made by glass grinder (42.22 *µ*m) and ultrasonicator (1.50 *µ*m). The same drug preparations were used as shown in Figure 3.

PK studies of WR299666 in two animal groups administered with particles in different sizes indicated that the values of the AUC and *C*_max⁡_ of WR299666 varied as a function of the particle size. The AUC and *C*_max⁡_ of the suspension with a particle size of 42.22 *μ*m, made by glass grinder, were significantly lower than those of the suspension with a particle size of 1.50 *μ*m, prepared using the sonicator (Table 5). The mean AUCs determined for the larger particle size of this compound were 387.44 ng·h/mL, whereas the mean values for the smaller particles were 718.63 ng·h/mL (*P*  value = 0.0132). The *C*_max⁡_ value determined for the suspension with larger particle size was 145.2 ng/mL, while the *C*_max⁡_ value of the smaller particle size suspension was 245.2 ng/mL (*P*  value = 0.0147). When the relative bioavailability of the small particle suspension of WR299666 in animals (1.50 *μ*m) was set as 100%, the relative bioavailability in mice treated with the larger particles (42.22 *μ*m) is only 53.7% (Figure 5).

### 3.5. Optimization of DQ Nanoparticle Formulations

**Figure 6:**
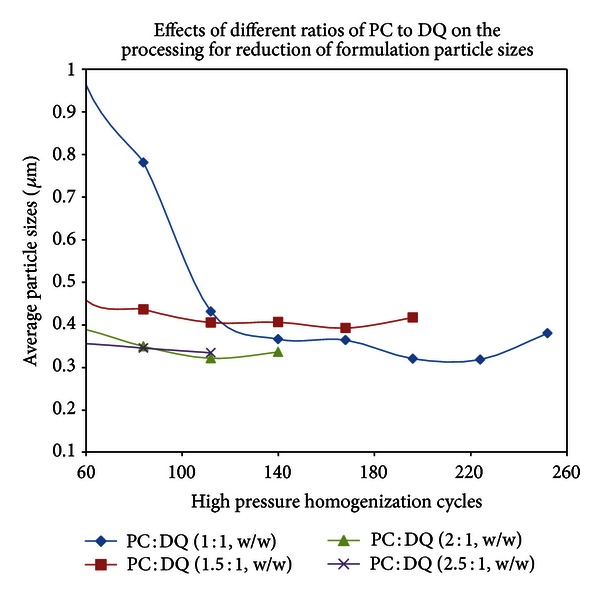
Different ratios of phosphatidylcholine (PC) to DQ were used in solid dispersion in the presence of polyvinylpyrrolidone 10 (PVP 10 : DQ = 6 : 1, w/w). After removal of solvents, dry film was suspended in water and subjected to particle size reduction by sonication and then HPH.

**Figure 7:**
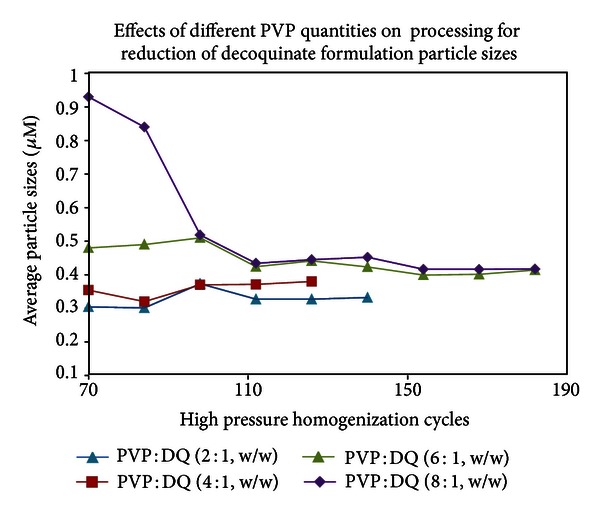
Different ratios of polyvinylpyrrolidone 10 (PVP 10) to DQ were chosen in the presence of phosphatidylcholine (PC : DQ = 2 : 1, w/w). After removal of solvents, the dry film was suspended in water and subjected to particle size reduction by sonication and then HPH.

**Figure 8:**
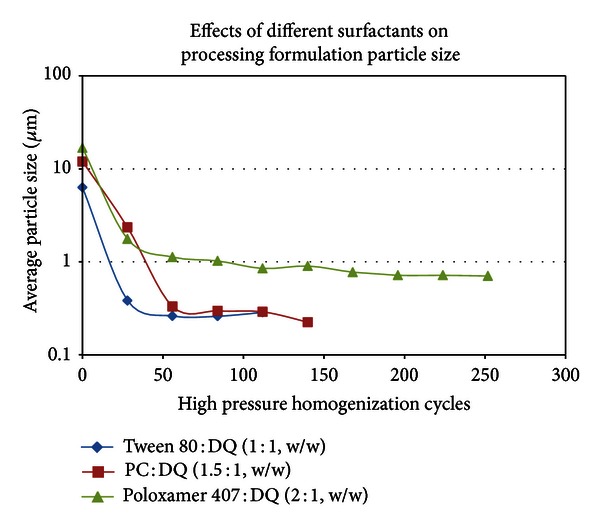
Different surfactants in the ratio relative to DQ as shown in the figure were applied for solid dispersion in the presence of polyvinylpyrrolidone 10 (PVP 10 : DQ = 3 : 1, w/w). After removal of solvents, the dry film was suspended in water and subjected to particle size reduction by sonication and then HPH.

A few surfactants were tested and validated to create a new DQ formulation. PC was found to be an excellent reagent for DQ formulation and different ratios of PC to DQ were applied in solid dispersion, followed by sonication and HPH (Figure 6). The optimized amount of PC relative to DQ was observed to be 1.5 : 1 to 2.5 : 1. Excessive amount of PC resulted in the formation of large particle size which was difficult to be reduced by sonication or HPH. Various polymers were tested to make DQ formulation. PVP, specifically PVP 10 (approximate MW: 10,000), however, was found to be superior to all other polymers tested such as PVP 40, PEGs, and a variety of celluloses. Different ratios of PVP 10 to DQ were examined in a formulation experiment (Figure 7) and the appropriate amounts of PVP 10 used were in a ratio of 3 : 1 or 4 : 1 to DQ. Different ratios of other surfactants such as Tween 80 and poloxamer 407 to DQ were also tested in the presence of PVP 10 to obtain an optimal amount of surfactant used in DQ formulation to generate nanoparticles by HPH. The selected DQ formulations with appropriated amount of PC, Tween 80, and poloxamer 407 were compared and shown in Figure 8. In our evaluation study for particle size stability, Tween 80 was found to be a very useful stabilizing agent in DQ formulation, whereas poloxamer 407 in DQ formulation, tended to produce larger and unstable particles (data not shown). 

## 4. Discussion

DQ has been used as an anticoccidial drug in the veterinary market for many years with no apparent adverse effects. Although this drug also has strong antimalarial activity, it has not been developed as an antimalarial drug for patients. The main reason could be the poor aqueous solubility of DQ, preventing adequate  *in vivo* delivery. Available information on the solubility of the drug [9] and our test results indicate DQ can be classified as practically insoluble or insoluble (0.06 *μ*g/mL in water). As a matter of fact, DQ suspended in HECT or in water had very poor absorption when dosed to animals. This was why a high dose (400 mg/kg) of DQ in microsuspension was given to animals in our PK studies. Lower dose of DQ in such preparation could lead to the failure to detect DQ in animal samples. Efforts in improving *in vivo* delivery to increase its bioavailability could be very challenging. 

In this study, evenly distributed microparticle suspension was made by directly grinding the drug mixed with small amount of polysaccharides (cellulose) and Tween 80 in distilled water by probe sonicator. However, this procedure failed and could not be used to generate DQ nanoparticles. Furthermore, DQ suspended directly in water or HECT was not suitable for particle size reduction by HPH. Thus, formulation of DQ with polymers and surfactants by using solid dispersion was adapted to create a suitable physical form of drug for particle size reduction. A polymer such as PVP 10 and surfactants such as PC or Tween 80 were necessary to formulate DQ for particle manipulation. By using this approach, DQ particle size could be reduced to 0.2–0.4 *μ*m. 

The selection of appropriate polymers and surfactants was also critical in the formation of nanoparticles. After many polymers were tested, PVP 10 was chosen for the formulation of DQ. Other polymers such as a variety of polyethylene glycols (PEG) and different kinds of celluloses were also tested in DQ formulation, and DQ became consistently aggregated during the process of sonication or HPH, even in the presence of appropriate amount of surfactants. PC and Tween 80 were two surfactants found to be appropriate components for DQ formulation and for further nanoparticle preparation. Other detergents such as poloxamer, sodium dodecyl sulfate, and sodium cholate were also tested and unsatisfactory results were obtained. For example, when poloxamer 407 was used in DQ formulation, the particle size reduction was difficult (Figure 8) and the generated nanoparticles tended to be larger (>0.5 *μ*m) and unstable due to particle agglomeration. 

Reduction in particle size has been shown to improve the efficiency of *in vivo* delivery and bioavailability of poorly soluble compounds. Sonication was commonly used as an initial step to bring down the particle size below 10 *μ*m and HPH used for further reduction of particle size. However, when DQ was directly suspended in aqueous medium or in inappropriate formulation with polymers such as PEG, the aggregates were formed during HPH procedure. Although HPH was shown to be the most effective technique to reduce particle size of DQ, an appropriate physical form of the drug needed to be created for HPH procedure. 

Four different methods, including glass grinding, homogenization with a slotted generator, probe and ultrasonication, and HPH, were applied to prepare WR299666 suspension. Manual force applied through a glass grinder, however, was ineffective in completely breaking apart large particles. HPH was found to be the far more effective technique in particle size reduction and was a relatively simple method to improve *in vivo* delivery and bioavailability of this drug. Ultrasonication treatment and other techniques used in this study were insufficient to produce nanoparticles for WR 299666. WR299666 nanoparticles prepared by HPH, while not used in PK study, were expected to have a more significant increase in bioavailability than a small micronized particle (1.5 *μ*m). 

The PK parameters and relative bioavailability of both DQ and WR299666 were measured in mice orally dosed with different preparations. The results show that DQ nanoparticle formulation produced significantly higher drug exposure levels in the plasma and in the liver than DQ microparticle suspension. Therefore bioavailability was increased with DQ nanoparticle given to animals. These results were not unexpected because a more rapid dissolution can occur with greater surface area of drug exposed. On the Noyes-Whitney equation [22] for dissolution of solids, the rate of dissolution is directly proportional to surface area. However, the relative distribution of DQ in the liver and in the blood was dramatically different in that DQ enrichment in the liver was much higher than that in the blood as summarized in Table 4. The ratios of the AUC in the plasma versus the AUC in the liver were 81.20 for DQ nanoparticle formulation and 20 for DQ microsuspension. Apparently the smaller particles resulted in significantly more accumulation of drug in the liver. These effects were significant and reproducible. Other tissues such as adipose tissue, kidneys, and skin, like the liver, have also been reported to have high DQ enrichment [23], but direct comparison of DQ distribution in these tissues relative to plasma has not been reported previously. 

Our data may have implication for DQ as a candidate antimalaria drug at the liver stage of malaria, which is an important early phase of malaria invasion of the human body. The high concentration of DQ distributed in the liver is very important due to the liver's role in the initiation of the complex *Plasmodium* life cycle. In experimental animals [24, 25], a female *Anopheles* mosquito infected with *P. berghei* parasites feeds on a mouse and injects the parasites in the form of sporozoites into the bloodstream. The sporozoites travel to the liver and invade liver cells. Over 2 days (47–52 hrs), the sporozoites grow, divide, and produce tens of thousands of haploid forms, called merozoites, per liver cell. This multiplication can result in millions of parasite-infected cells in the host bloodstream, leading to illness and death within 7 days. The analysis of antimalarial drug efficacy against liver-stage malaria is therefore much more complex than that of the efficacy against blood-stage parasites. Surprisingly, the PK data in our study showed that in contrast to DQ microparticle suspension, DQ nanoparticle suspension produced significantly higher drug exposure levels in the liver than those in the plasma. 

Atovaquone is the structural analog of DQ widely used in malaria prevention. Compared to atovaquone, DQ has equivalent or higher *in vitro* potency against malaria [7, 8] but has limited cross-resistance to a panel of atovaquone-resistant parasites [7]. Our DQ nanoformulation may change the scope of the potential use of this compound. This strategy may lead to further exploitation of DQ as an antimalarial candidate, especially for malaria liver stage inhibition. 

## 5. Conclusions

DQ nanoparticle formulations were made by choosing appropriate formulation components and particle size reduction techniques. The particles in the range of 200–400 nm were shown to have significantly increased bioavailability compared to its original drug form with micro-sized particles. WR299666 nanoparticles produced by a simple procedure were also expected to have increased bioavailability. The enhanced bioavailability should favor the *in vivo* antimalarial activity of these agents. 

## Figures and Tables

**Table 1:** Particle size (*μ*m) measurements of DQ in water or HECT suspension stored at 4°C.

Stability duration	Methods		
	HG	US	SD plus HPH
Week 1	99.23	36.62	0.401
Week 2	101.33	36.97	0.384
Week 3	105.56	36.92	0.377
Mean	102.04	36.88	0.392

SD: solid dispersion; HG: homogenizer with generator; US: ultra-sonicator; HPH: high pressure homogenization.

**Table 2:** Particle size (*μ*m) measurements of W299666 in water or HECT suspension stored at 4°C.

Stability duration	Methods			
	Grinder	HG	US	HPH
Week 1	42.54	66.31	1.52	0.376
Week 2	41.07	71.85	1.58	0.397
Week 3	43.05	47.49	1.41	0.445
Mean	42.22	61.88	1.50	0.406

HG: homogenizer with generator; US: ultra-sonicator; HPH: high pressure homogenization.

**Table 3:** Evaluations* of pharmacokinetics and drug distribution in liver of decoquinate (DQ) at dose of 80 mg/kg with various formulations in male ICR mice (*n* = 5) following single intragastric administration.

PK parameters	Decoquinate in plasma		Decoquinate in liver	
	Microsuspension (36.88 *μ*m)	Nanosuspension (0.39 *μ*m)	Microsuspension (36.88 *μ*m)	Nanosuspension (0.39 *μ*m)
*C*_max⁡_ (ng/mL or g)	6.1 ± 0.7	25.3 ± 2.9	93.4 ± 9.7	407.3 ± 54.4
*T*_max⁡_ (hr)	6.50 ± 2.12	3.66 ± 1.16	6.50 ± 2.12	3.00 ± 2.00
AUC_last_ (ng·h/mL or g)	46.2 ± 2.5	367.9 ± 26.8	1057.4 ± 184.8	5768.7 ± 1104.8
AUC_inf._ (ng·h/mL or g)	51.4 ± 6.2	795.5 ± 36.19	1096.2 ± 170.3	6055.5 ± 1116.1
*t*_1/2_ elimination (*β*, h)	8.17 ± 2.59	23.93 ± 14.20	5.12 ± 0.40	6.36 ± 1.43
Vz/*F* (liter/kg)	3652.3 ± 732.4	925.7 ± 505.3	—	—
CL/*F* (liter/hr/kg)	313.6 ± 37.6	117.8 ± 58.9	—	—
MRT (h)	7.47 ± 0.79	4.51 ± 0.71	7.16 ± 0.42	7.51 ± 0.58

Relative bioavailability (%)	6.77	100	—	—
Drug distribution in liver	—	—	18.1	100

*The data was fitted with Phoenix/WinNonlin (V6.1). PK: pharmacokinetics; MRT: mean residence time.

**Table 4:** Summary of orally dosed DQ distribution in the liver and in the blood.

PK values and	Nanoparticle (0.39 *μ*m)		Microsuspension (36.88 *μ*m)	
Ratios of liver/plasma	PO 80 mg/kg	PO 80 mg/kg	PO 400 mg/kg	PO 400 mg/kg
AUC liver (ng·h/mL)	6055.50		1096.20	
*C*_max⁡_ liver (ng/mL or g)		407.30		93.40
AUC plasma (ng·h/mL)	795.50		51.40	
*C*_max⁡_ plasma (ng/mL or g)		25.30		6.10

Ratio of AUC liver/AUC plasma	81.20		21.30	
Ratio of *C*_max⁡_ liver/*C*_max⁡_ plasma		16.10		15.30

Data are derived from Table 3. DQ: decoquinate; PO: oral dose; AUC: area under the curve; *C*_max⁡_: maximal concentration; DQ was orally dosed at 80 mg/kg for nanoparticle formulations and at 400 mg/kg for microsuspensions.

**Table 5:** Evaluations* of pharmacokinetics of WR299666 suspended in HECT at dose of 50 mg/kg following single oral administration in male ICR mice (*n* = 5).

PK Parameters	WR299666 in Plasma	
	Microsuspension (42.22 *μ*m)	Microsuspension (1.50 *μ*m)
*C*_max⁡_ (ng/mL)	145.20 ± 9.40	245.20 ± 71.10
*T*_max⁡_ (hr)	0.70 ± 0.08	0.79 ± 0.15
AUC_last_ (ng · h/mL)	387.40 ± 35.90	718.60 ± 131.30
AUC_inf._ (ng · h/mL)	405.10 ± 36.60	732.40 ± 127.00
*t*_1/2_ elimination (*β*, h)	9.00 ± 0.90	7.94 ± 0.52
Vz/*F* (liter/kg)	613.70 ± 39.40	267.80 ± 79.20
CL/*F* (liter/hr/kg)	1.97 ± 0.19	1.24 ± 0.27
MRT (h)	5.81 ± 0.31	1.22 ± 0.20

Relative bioavailability (%)	53.70	100.00

*The data was fitted with Phoenix/Win Nonlin (V6.1). PK: pharmacokinetics; MRT: mean residence time.

## Abbreviations

DQ: Decoquinate
HPH: High pressure homogenization
PK: Pharmacokinetics
HECT: 0.5% (w/v) hydroxyethyl cellulose and 0.2% (v/v) Tween 80.
